# Infectious Pancreatic Necrosis Virus Causing Clinical and Subclinical Infections in Atlantic Salmon Have Different Genetic Fingerprints

**DOI:** 10.3389/fmicb.2016.01393

**Published:** 2016-08-31

**Authors:** Stephen Mutoloki, Trude B. Jøssund, Gordon Ritchie, Hetron M. Munang'andu, Øystein Evensen

**Affiliations:** ^1^Faculty of Veterinary Medicine and Biosciences, Norwegian University of Life SciencesOslo, Norway; ^2^Marine Harvest ASABergen, Norway

**Keywords:** salmon, infectious pancreatic necrosis virus, genotype, clinical, subclinical

## Abstract

Infectious pancreatic necrosis virus (IPNV) is the causative agent of IPN, an important disease of salmonids. IPNV infections result in either sub-clinical or overt disease and the basis of this difference is not well-understood. The objective of the present study was to determine the VP2 gene of the virus associated with the different forms of clinical manifestation. Groups of Atlantic salmon (*Salmo salar* L.) reared in farms located in different IPN disease pressures were monitored from brood stock until grow-out over a 3 year period. Hatcheries A1 and B1 as well as cooperating seawater farms were located in a low disease risk area while hatcheries A2 and B2 as well as their cooperating seawater farms were in high IPN risk areas. Samples including eggs, milt, whole fry, kidney depending on the stage of production were collected during outbreaks or in apparently healthy populations where no outbreaks occurred. The virus was re-isolated in CHSE cells and the VP2 gene amplified by RT-PCR followed by sequencing. During the freshwater stage, there were no disease outbreaks at hatcheries A1, A2, and B1 (except in one fish group that originated from hatchery B2), although IPNV was isolated from some of the fish groups at all 3 hatcheries. By contrast, all fish groups at hatchery B2 suffered IPN outbreaks. In seawater, only groups of fish originating from hatchery A1 had no IPN outbreaks albeit virus being isolated from the fish. On the other hand, fish originating from hatcheries A2, B1, and B2 experienced outbreaks in seawater. The VP2 amino acid fingerprint of the virus associated with subclinical infections from A1 and co-operating seawater sites was V64A137P217T221A247N252S281D282E319. By contrast, all virus isolates associated with clinical infections had the motif I64T137T217A221T247V252T281N282A319, where underlined amino acids represent the avirulent and highly virulent motif, respectively. Phylogenetic analysis of amino acid sequences showed 2 clades, one of isolates associated with subclinical infections (from A1 and cooperating seawater farms) and the other of isolates from fish with overt disease (all other sites). Furthermore, the clustering pattern of isolates suggests more circulation of virus within fish groups rather than between them.

## Introduction

Infectious pancreatic necrosis (IPN) remains one of the most important viral diseases of farmed salmonids in Norway. It is caused by the IPN virus (IPNV), a non-enveloped, double stranded RNA virus. The virus is a prototype in the genus *Aquabirnavirus* and belongs to the family *Birnaviridae*. It affects Atlantic salmon at all stages of production especially at start-feeding of fry, in fingerlings and parr during the fresh water stage as well as in post-smolts 3–4 weeks following sea water transfer (Roberts and Pearson, 2005).

In general, IPNV transmission occurs horizontally (Gregory et al., 2003). Although vertical transmission has been demonstrated in rainbow trout (*Oncorhynchus mykiss*; Dorson and Torchy, 1985), it has not been definitively proven in Atlantic salmon (*Salmo salar* L.). Nevertheless, adsorption of the virus to the surface membrane of sperms and egg fluid would be one method by which it occurs (Wolf et al., 1963; Mulcahy and Pascho, 1984; Reno, 1999; Smail and Munro, 2008). Outbreaks of IPN in fry at start-feeding are thought to be as a result of this method of transmission (Roberts and Pearson, 2005). Survivors of IPNV infection become persistently infected and are sources of infection to naïve fish (Roberts and Pearson, 2005). The ability of the virus to survive in the environment and in alternative hosts (Mulcahy and Pascho, 1984; Rimstad, 2003; Gregory et al., 2007) ensures that the infection is perpetuated through subsequent stocks of fish at particular sites.

Predisposing factors for disease outbreaks are not known in detail although host-related, virus-associated, and environment factors are all important. For hosts, differences in the susceptibility between fish families (Okamoto et al., 1993) point to genetic variation playing a role. This has recently been demonstrated by the introduction of QTL fish that has shown resistance against the disease (Houston et al., 2008). For the virus, previous studies where Norwegian IPNV isolates obtained from Atlantic salmon during field outbreaks were used to experimentally challenge fish showed that certain amino acids in the capsid protein are associated with virulence (Santi et al., 2004). By using reverse genetics, these amino acids were mapped to positions 217, and 221, with highly virulent isolates encoding the T_217_A_221_ motif while avirulent isolates had P_217_T_221_ (Song et al., 2005). Despite this knowledge, traits of IPNV associated with clinical or subclinical infections of fish in fresh and seawater under field conditions have remained unclearly documented, with some authors reporting mortalities associated with IPNV having the P_217_T_221_ motif (Bain et al., 2008). The purpose of the present study was to investigate genetic fingerprints of field strains of IPNV associated with clinical or asymptomatic disease. A traceback study was used to determine whether outbreaks of IPN or absences thereof, in the field, were linked to specific amino acids on the major capsid protein of the virus. IPNV from production lines (broodstations, hatcheries, seawater sites) reported to have had major or minor IPN outbreaks were targeted and genetic sequences of the capsid protein (VP2) of IPNV from infected fish were examined. The VP2 protein is the major structural protein encoded by the large open reading frame of segment A (Macdonald and Dobos, 1981; Duncan et al., 1987) and comprises proteins 1–442 of the polypeptide (Galloux et al., 2004). It was preferred for this analysis because of its implication in virulence, serotype specificity, and immunogenicity (Heppell et al., 1993; Frost et al., 1995; Bruslind and Reno, 2000; Shivappa et al., 2004; Song et al., 2005).

## Materials and methods

This study was approved by the Norwegian Animal Research Authority. Prior to sampling, the fish was anesthetized with Finquel® (100 mg/L) in order to prevent suffering.

### Study design and animals used

The present study was undertaken in the mid Northwestern coast of Norway over a 3-year period. The fish farms targeted were categorized as either high or low IPN risk on the basis of the number of outbreaks that had occurred during the previous 3 years prior to the onset of the present study. Fish farms that had had at least three outbreaks over 3 seasons prior to onset of the study were categorized as being in the high-risk group while those with 0–2 outbreaks were considered as low risk. To a large extent, samples were collected from each developmental stage in different groups of fish. Here “group” implies fish hatching from one batch of eggs and subjected to the same production treatment until grow-out at sea. Hatcheries and freshwater sites are used interchangeably. Clinical infections refer to fish exhibiting signs of IPN and following sampling, were confirmed diseased by the National Veterinary Institute in Norway.

**Figure 1:**
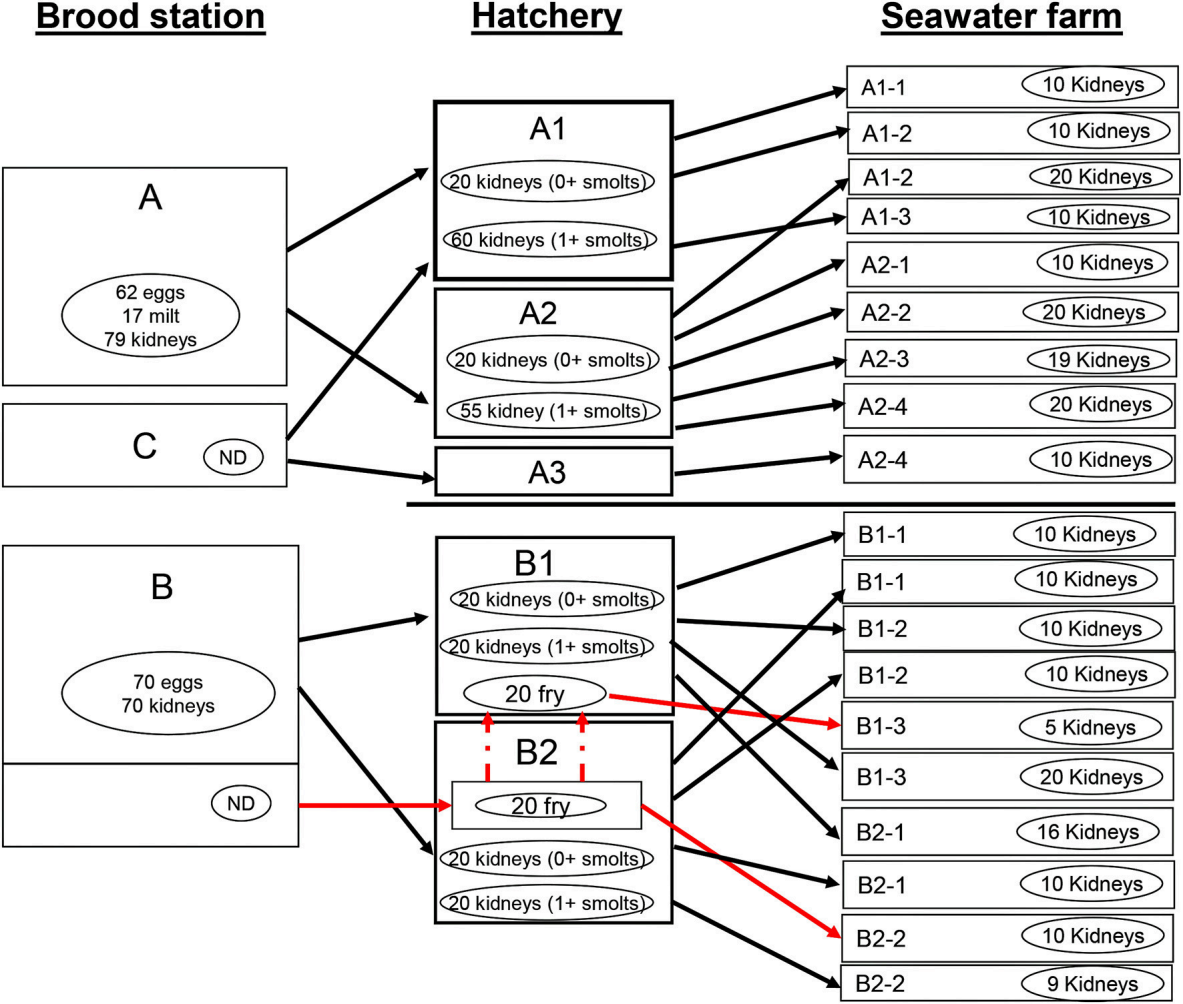
**Study design and flow of breeding materials from fertilized eggs and fish in broodstations to grow out farms**. Type of samples and the numbers collected at each stage are provided for each group of fish. Each rectangle represents an independent fish group and arrows link the groups at different stages. ND, no samples collected; red dotted line, infected fry transfer at freshwater stage.

At the start of the study, hatcheries A1 and B1 and co-operating seawater farms, i.e., A1-1 to A1-3 and B1-1 to B1-3, respectively were designated as being in the low IPN risk category (Figure 1). Hatcheries A2 and B2 as well as the co-operating seawater farms were in the high-risk category. One fish group sampled at one of the seawater farms (A2-4, Figure 1) originated from hatchery A3 that was not part of the hatcheries included in this study.

The main source of fertilized eggs for the hatcheries (A1 and A2) and (B1 and B2) were brood stations A and B, respectively (Figure 1). In addition, hatcheries A1 received fertilized eggs from another brood station C.

Brood stations A and C belonged to the same organization and therefore were subject to similar management practices while brood station B belonged to a different company and had a different management practice. Similarly, hatcheries/freshwater sites A1 and A2 and seawater farms A1-1 to A1-3 and A2-1 to A2-4 belonged to the same organization as brood stations A and C. This cooperation is the basis for grouping hatcheries A1 with A2 and their cooperating seawater farms in Figure 1. Hatcheries B1 and B2 belonged to another company cooperating with corresponding seawater farms designated B. Finally, all the fish in this study were reared according to standard fish farming practices in Norway.

During the freshwater stage, fingerlings, and parr were sorted according to class sizes and reared as units identifiable as groups. This was done several times at all sites. The fish were cultivated both as in-season (S1) and also out of season (S0). Accordingly, smolts were put to sea twice, as 1+ smolts (after 18 months in fresh water) and 0+ smolts (10 months in fresh water), respectively.

### Sample collections

Samples were collected at different stages of production as described below.

#### Brood stations

Following stripping of eggs and milt from breeders for each batch, samples of eggs and milt were taken. Breeders were sacrificed prior to sampling of head kidney. At brood station A, the batches from which samples were collected were chosen randomly. The numbers of samples are shown in Figure 1. At brood station B, only eggs as well as organ samples from the donors were collected. No samples were collected from stations C.

The handling of samples following collection was as follows: eggs and milt were frozen immediately after collection and transported to the Norwegian University of Life Sciences; the kidney samples from brood fish were preserved in serum-free transport media consisting of Leibovitz's L-15 medium (Sigma Aldrich) supplemented with 50 μg ml^−1^ Gentamycin.

#### Hatcheries/freshwater stage

Sampling was done during IPN disease outbreaks, targeting moribund or dying fish. As shown in the study design (Figure 1), samples from freshwater sites were collected from the same batches as eggs and milt were sampled. Fry were collected whole while kidney samples were collected from larger fish and preserved in transport medium during transportation. In the absence of outbreaks, randomly selected parr were sampled. As already stated, the fish were sorted several times at this stage and therefore mixed with fish from different units within the same group thereby expanding/diluting the pool.

#### Seawater farms

For clarity, the first two letters in the name of the seawater farms are derived from the hatchery that they primarily co-operated with (Figure 1). Each farm should be viewed as an independent entity.

The same procedure as in freshwater was used to sample post-smolts/growers in seawater (Figure 1) targeting the same groups. The IPN disease in all outbreaks was provisionally diagnosed on the basis of clinical signs and confirmed by immunohistochemistry at the National Veterinary Institute (Trondheim).

### Virus isolation by cell culture

Chinook salmon embryo cells (CHSE-214, ATCC CRL-1681) were used for screening of IPNV. The cells were incubated at 20°C and were routinely maintained in Leibovitz's L-15 medium supplemented with 5% FBS and 50 μl^−1^ gentamycin.

To prepare the samples for inoculation, Atlantic salmon eggs were homogenized in a stomacher (IUL Instrument) for 1 min, diluted by adding an equal amount (w/v) of transport medium followed by centrifugation at 2500 × g for 10 min at 4°C. Thereafter the supernatant was harvested and stored at −70°C until required. Kidney samples were homogenized the same way in serum-free L-15 medium supplemented with 50 μl^−1^ Gentamycin (1:10 w/v). The milt was prepared by centrifugation at 2500 rpm for 10 min at 4°C, harvesting the supernatant in an equal volume of transport medium.

The samples were inoculated individually, in two dilutions (1:10 and 1:100) on confluent CHSE cells seeded on 24 wells plates (Sigma). The presence or absence of virus was established by the presence/absence of cytopathic effect (CPE) after two passages. Three randomly selected positive samples per group from each site were then used as starting material for genotyping of IPNV-VP2 gene.

### RNA extraction, sequencing, and genotyping

The cell culture supernatants were first clarified by centrifugation at 2500 × g for 10 min at 4°C. The viral RNA isolation kit (Qiagen) was used to isolate viral RNA starting with 140 μl of the supernatant, according to the manufacturer's instructions. Amplification of VP2 gene was done by PCR using gene-specific primers A-sp1-24F and A-sp-1696R (Table 1) in a one-step RT-PCR (Qiagen). PCR products were then separated by using 1% Agarose gel electrophoresis and extracted using the QIAquick Gel Extraction Kit (Qiagen). Sequencing was done by direct sequencing of PCR products on a commercial basis at MWG Biotech (Germany) using 3 primers A-sp59F, A-sp500F, and A-sp1689R (Table 1) as previously done (Santi et al., 2004). The sequence data was assembled using Vector NTI software (Invitrogen). Analysis and translation of sequences was done by using SDSC Biology workbench 3.2 (workbench.sdsc.edu). The CLC Main Workbench 6.0 (www.clcbio.com) and Mega7 software (Kumar et al., 2016) were used for sequence alignment and phylogenetic and evolutionary analyses. The phylogenetic tree of the major capsid protein was inferred by the Maximum Likelihood method, bootstrapped 1000 times based on the JTT+G matrix-based model (Jones et al., 1992).

**Table 1:** **Gene-specific primers for infectious pancreatic necrosis virus used in the present study**.

**Name**	**Sequence**	**Nucleotide no**.
A-sp1-24F	GGAAAGAGAGTTTCAACGTTAGTG	1–24
A-sp1696R	GGACTCCAGCCTGTTCTTGAG	1696–1676
A-sp59F	TCTCCGTCGATGGCGAAAG	59–77
A-sp500F	GAGTCACAGTCCTGAATC	500–517
A-sp1689R	AGCCTGTTCTTGAGGGCTC	1689–1671

### Structural analysis of the VP2 capsid

To gain insight into the structural layout of amino acids influencing the antigenic variabilities detected from the study sites, the SWISS model workspace (Arnold et al., 2006) and Pymol version 99 (PyMol, 2012) were used to align the sequences and to address the structural layout of these resides as well as to determine their potential influence on the clinical and subclinical conditions observed from the study sites, respectively.

## Results

A total of 910 samples including kidneys, eggs, milt, and whole fry from 3 brood stations, 5 hatcheries, and 12 sea water sites were processed throughout the study period. Tissue samples collected from brood fish, eggs, and milt at brood stations A and B yielded negative IPNV isolation. As already mentioned, no samples were collected from brood fish at station C. IPNV was isolated from fish in both hatcheries and seawater sites. The VP2 gene of the virus was amplified by PCR and sequenced. The sequence information as well as the source of samples is presented in Table 2.

**Table 2:** **Samples from which infectious pancreatic virus was isolated in the present study**.

**Sample**	**Source**	**Fish type**	**Genbank accession no**.
A1_32	A1	Parr	KX355261
B1-3_36	B1-3	Smolt	KX355260
A1-3_53	A1-3	Smolt	KX355259
A1-1_9	A1-1	Smolt	KX355258
A1_51	A1	Smolt	KX355257
A1-2_4	A1-2	Smolt	KX355256
A1_48	A1	Parr	KX355255
A1-3_54	A1-3	Smolt	KX355254
A1-3_28	A1-3	Smolt	KX355253
A1-2_6	A1-2	Smolt	KX355252
B1-3_37	B1-3	Smolt	KX355251
A2-1_30	A2-1	Smolt	KX355250
B2-1_5	B2-1	Smolt	KX355249
A2-4_41	A2-4	Smolt	KX355248
A2-4_40	A2-4	Smolt	KX355247
A1-2_1	A1-2	Smolt	KX355246
A2-4_46	A2-4	Smolt	KX355245
B2-2_39	B2-2	Smolt	KX355244
A2-4_38	A2-4	Smolt	KX355243
B1-1_43	B1-1	Smolt	KX355242
B1_47	B1	Fry	KX355241
B2-2_33	B2-2	Smolt	KX355240
B1-1_44	B1-1	Smolt	KX355239
B1-3_8	B1-3	Smolt	KX355238
A2_29	A2	Fry	KX355237
A2-2_45	A2-2	Smolt	KX355236
B2-2_42	B2-2	Smolt	KX355235
B2-2_34	B2-2	Smolt	KX355234
B1-2_31	B1-2	Smolt	KX355233
B2_52	B2	Parr	KX355232
B2_50	B2	Parr	KX355231
B2_26	B2	Fry	KX355230
B2_27	B2	Fry	KX355229
B1-3_7	B1-3	Smolt	KX355228
B1-3_35	B1-3	Smolt	KX355227
B1-3_3	B1-3	Smolt	KX355226
A1_2	A1	Parr	KX355225
B1_49	B1	Fry	KX355224
B2-2_18	B2-2	Smolt	KX355223
B2-2_15	B2-2	Smolt	KX355222
A1-2_14	A1-2	Smolt	KX355221
A2-3_13	A2-3	Smolt	KX355220
A1-2_12	A1-2	Smolt	KX355219
A1-2_11	A1-2	Smolt	KX355218
B1-1_16	B1-1	Smolt	KX355217
B1-1_17	B1-1	Smolt	KX355216
B1-3_10	B1-3	Smolt	KX355215

### Low IPN disease risk category

#### Hatchery A1 and co-operating seawater farms A1-1 to A1-3

**Figure 2:**
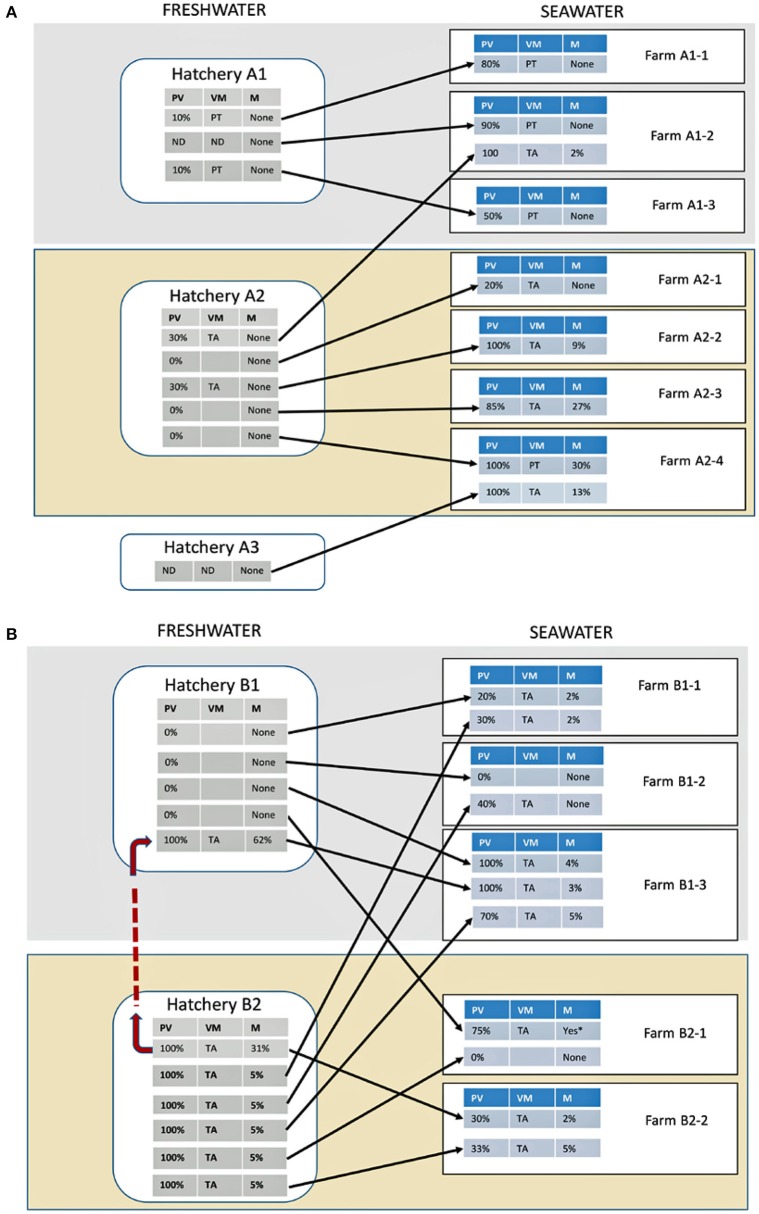
**Infectious pancreatic necrosis virus (IPNV) infections and mortalities in different groups of Atlantic salmon in hatcheries and seawater farms**. **(A)** Hatcheries and seawater sites that received breeding materials primarily from brood station A and **(B)** Hatcheries and seawater sites that received breeding materials primarily from brood station B. Each block (rectangle) within a hatchery/farm represents an independent fish group and arrows connect the groups at different stages. PV, % prevalence of infection; VM, virulence motif represented by amino acid residues 217 and 221 of the VP2 protein; M, mortality due to IPNV; ND, No samples collected; red dotted line, infected fry transfer at freshwater stage; ^*^Mortality figures not available. Gray and yellow background colors represent the primary setup of hatcheries and seawater farms cooperation.

No disease outbreaks were observed at hatchery A1 although IPNV was isolated from apparently healthy fish (Figure 2A). The prevalence of infection in fish was estimated at 10% and the virulence motif (Santi et al., 2004) was found to be P_217_T_221_ (PT) in all cases.

No outbreaks were observed at seawater farms A1-1 to A1-3 receiving smolts from hatchery A1. IPNV was isolated from apparently healthy individuals in seawater but this time the prevalence of infection was 50–90% (Figure 2A). Seawater farm A1-2 received smolts from hatchery A2 in addition to that from A1 and it is the group from A2 that an IPN outbreak was experienced. The prevalence of infection in this group was 100% and the motif of the virus was T_217_A_22_ (TA), similar to that found at hatchery A2.

#### Hatchery B1 and co-operation seawater sites and B1-1 to B1-3

At hatchery B1, no disease outbreaks were observed except in one fish group (Figure 2B). The outbreak was in a group of fry that had been transferred from a high-risk hatchery B2 where the mortality was 31%. The mortality in fry at B1 was 62% and the prevalence of infection 100%. The virulence motif was TA, similar to that found at the hatchery B2.

For seawater farms B1-1 to B1-3, IPN outbreaks were observed in all except one farm (B1-2), irrespective of whether or not IPNV had been isolated from the fish group during the freshwater stage. The prevalence of infection in all groups ranged from 20 to 100% while mortalities were between 2 and 5% (Figure 2B). The virulence motif of the virus was TA, similar to the strain isolated from the fish groups at hatcheries B1 and B2 where outbreaks were observed. Seawater farms B1-1 to B1-3 received smolts from B2 in addition to the supply from B1 (Figure 2B). Surprisingly, only one farm (B1-2) as mentioned did not experience any IPN outbreak.

### High IPN disease risk category

#### Hatchery A2 and co-operating seawater farms A2-1 to A2-4

Although hatchery A2 was categorized as high risk, there were in fact no disease outbreaks during the study period. In two fish groups however, ~30% of the fish sampled were infected with IPNV, with virulence motif of TA while no IPNV was isolated from 3 out of the 5 groups (Figure 2A).

When smolts were transferred to seawater farms, IPNV was isolated from all fish groups and outbreaks were experienced in all except one group at farm A2-1. In this group, the prevalence of infection was 20% as opposed to the others where it ranged from 85 to 100% (Figure 2A). The virulence motif of the virus in all cases was TA, similar to that of the hatchery A2 where the fish originated. An extra group of fish from an external hatchery (A3) was included in the sampling plan at farm A2-4. This group was also infected with the TA variant of IPNV and had a prevalence of 100 and 13% mortality.

#### Hatchery B2 and co-operating seawater farms B2-1 and B2-2

The hatchery B2 experienced the highest number of outbreaks, mortalities and prevalence. All fish groups had IPN outbreaks. The prevalence of infection in all groups was 100% and the virulence motif was TA while mortalities ranged from 5 to 31% (Figure 2B).

Hatchery B2 co-operated primarily with only two seawater farms (B2-1 and B2-2) targeted in this study and totally 4 fish groups at these sites were examined. One of the sites (B2-1) received smolts from B1 in addition (Figure 2B). These groups of fish became infected at sea and the prevalence was 75% (Figure 2B). Although there was an outbreak in this group, mortality figures were unfortunately not available.

At farm B2-2, the two groups examined had 30 and 33% IPNV prevalence and 2 and 5% mortalities, respectively. The variant of the virus was TA.

### Genomic analysis of the VP2 genes from different isolates

Translated amino acid sequence alignments of virus isolates in this study revealed that isolates associated with clinical and subclinical infections had different genetic fingerprints in the capsid protein (VP2). Isolates associated with subclinical infections had a consensus amino acid motif of V_64_A_137_P_217_T_221_A_247_N_252_S_281_D_282_E_319_ while those from fish with clinical had I_64_T_137_T_217_A_221_T_247_V_252_T_281_N_282_A_319._

**Figure 3:**
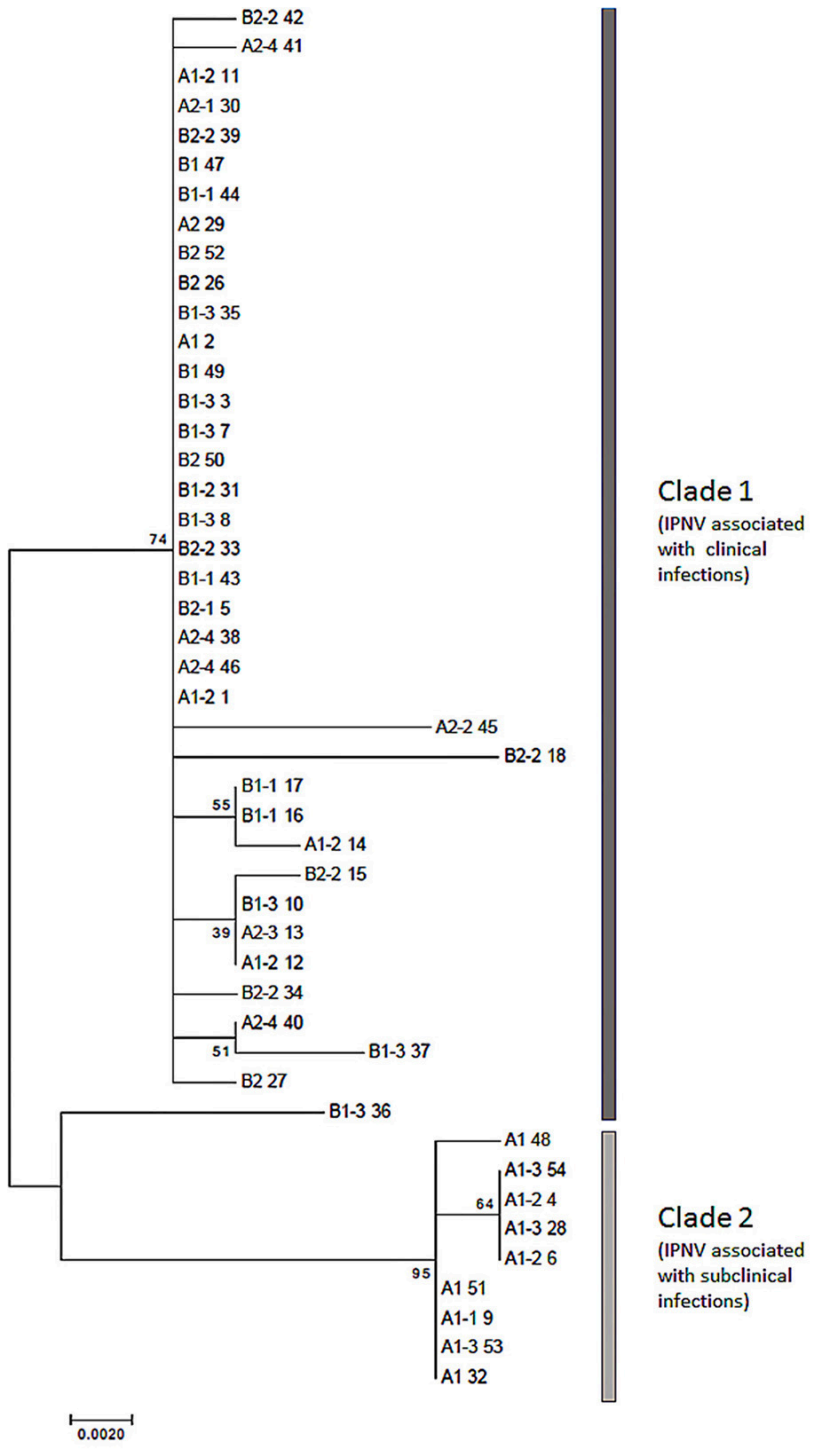
**Phylogenetic tree of the major capsid protein of IPNV isolated from Atlantic salmon in the present study**. The tree was inferred by the Maximum Likelihood method based on the JTT matrix-based model (Jones et al., 1992). Evolutionary analyses were done using translated protein sequences in MEGA7 (Kumar et al., 2016). Key: Isolate names are designed to show the source of isolation while the number following the space is the unique sequence identifier, e.g., A1 3 = isolate from hatchery A1 and 3 is the unique sequence identifier, A1-1 44 represents isolate from SW farm 1 and 44 is the unique identifier for this sequence; Gray lines represent clades.

Phylogenetically, the isolates clustered into 2 main clades (Figure 3). Clade 1 constituted isolates associated with overt disease in Atlantic salmon. By contrast, Clade 2 was composed of isolates from hatchery A1 and associated seawater sites, of fish that were infected with the isolates inducing subclinical forms.

### Structural analysis of VP2 capsid of different isolates

**Figure 4:**
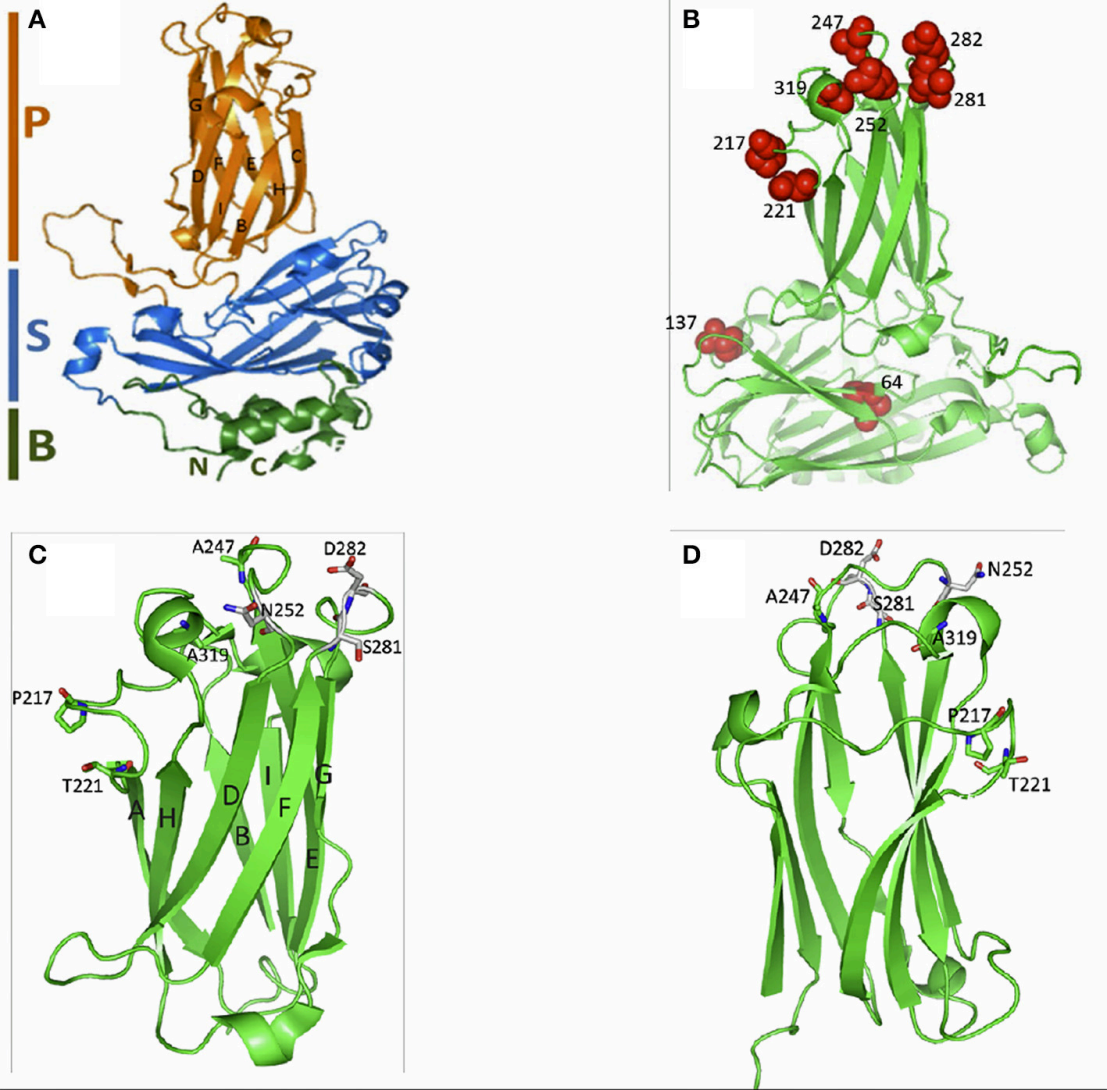
**Subviral particle (SVP) of the VP2 capsid for IPNV**. **(A)** Shows the SVP divided into the P-domain (magenta) made of the β-sheet that supports the surface loops that form the hypervariable region (HVR), S-domain (blue) that form the shell of the capsid, and the B-domain (green) that form of the base made of the N- and C-termini. **(B)** Shows the position of the residues linked to clinical and subclinical forms of IPNV infection (red balls). Note that positions V64I and A137T are located in the B-domain while T217P and A221T on loop P_BC_, residues T247A and V252N on P_DE_, residues T281S and N282D on P_FG_ and A319E on P_HI_ in the surface loops of the HVR in the P-domain. **(C)** Shows the P-domain having the β-sheets supporting the surface loops of the HVR. The structural layout of residues T217, A221T, T247, N252, S281, D282 and A319 are shown as sticks projecting out of the surface loops of the HVR. **(D)** Shows the P-domain at 1800 turn of **(C)** All figures were generated in Pymol v99 (PyMol, 2012) using the 3IDE template (Coulibaly et al., 2010) on the SWISS model workspace (Arnold et al., 2006).

Figure 4 shows the positions of amino acid residues on the VP2 subviral particle (SVP). Note that residues V64I and A137T are located in the B-domain of the SVP while residues T_217_P and A_221_T are on loop P_BC_, T_247_A, and V_252_N are on P_DE_, T_281_S, and N_282_D on P_FG_ while A_319_E is on P_HI_ of the hypervariable region (HVR) in the P-domain of the VP2 capsid.

## Discussion

The findings of the present study demonstrate that IPNV causing clinical and subclinical forms of infections under field conditions have specific genetic fingerprints. Subclinical infections were associated with a V_64_A_137_P_217_T_221_A_247_N_252_S_281_D_282_E_319_ (PT) fingerprint on the major capsid protein (VP2) while isolates associated with overt disease had the fingerprint I_64_T_137_T_217_A_221_T_247_V_252_T_281_N_282_A_319_ (TA). Fish at hatchery A1 and corresponding seawater farms A1-1 to A1-3 that received smolts from this hatchery were all infected with the former, and no mortalities were experienced irrespective of the degree of prevalence of infection (Figure 2A). On the other hand, all fish groups with IPN disease outbreaks were infected with the TA variant (Figure 2). Previous studies have shown that virulent and avirulent strains of IPNV have specific amino acids in the VP2 protein represented by motifs T_217_A_221_ and P_217_T_221_, respectively (Santi et al., 2004; Song et al., 2005). These two positions are part of the genetic fingerprint reported here and our findings are consistent with these reports.

The most affected hatchery with IPN in this study was B2 where virtually all groups examined experienced outbreaks (Figure 2). Mortalities due to IPN persisted in most of the groups following transfer to seawater suggesting that infections were carried along as previously reported by others (Roberts and Pearson, 2005). Interestingly, the prevalence of infection in all groups decreased from fresh to seawater. In two groups (B1-2 and B2-1), no mortalities were experienced in seawater while in one (B2-1) even the virus could not be detected suggesting that the fish has shed it off completely. It is believed that fish develop resistance to IPNV infection with age and our present findings support this view (Munro and Midtlyng, 2011). The differences in the resistance acquired between groups in this study suggests that other factors, such as the environment also contributes to the outcome of infections between sites (Munro and Midtlyng, 2011).

At hatcheries A2 and B1, most of the fish groups were not infected with IPNV and yet when transferred to sea, almost all groups developed IPN with high prevalence (Figure 2). Furthermore, fish groups from hatchery A2 experienced high mortalities (9–30%) following seawater transfer while only 2–4% mortalities were observed in groups from B1. The patterns of infection of fish following seawater transfer points toward seawater sites being reservoirs of IPNV (Murray et al., 2003) while differences in mortalities suggest that other factors such as environment play a role in the severity of infections and further studies should be conducted to elucidate these.

Being an RNA virus, IPNV lacks the proofreading ability inherent of DNA polymerases and is thus prone to mutations during replication. Hsu and colleagues showed that this virus exists as a quasi-species (Hsu et al., 1995). This is also reflected in the heterogeneity of the isolates obtained in this study (Figure 3). Inadvertently, the different variants of IPNV replication have different fitnesses. One of the traits of the highly virulent (TA) isolate is the superior replication capacity over the less virulent (TT) mutant (Gadan et al., 2013), although this was less so when compared with the PT isolate (Gamil et al., 2015). In another study, the TA variant showed superior capacity to lyse cells compared to the PT variant (Gamil et al., 2015). In support of this observation, the prevalence of the TA isolates in this study compared to their PT counterparts in naïve fish (hatcheries A2, B1, and B2 vs. A1, Figure 2) was higher. Although the amount of virus in individual fish was not measured, it is tempting to speculate that the ability of the TA isolates to spread between fish is related to their efficiency in cell lysis. The dynamics of virus infections in fish during the seawater stage gives a less obvious pattern given that the source of infection could have been either during the freshwater stage, seawater or both. What is interesting though was the general trend whereby low prevalence during the freshwater stage resulted in high prevalence during the seawater stage and vice versa (Figure 2). To some extent, the levels of mortality also reflect this observation. Whether low to high prevalence in fish from fresh- to seawater is a function of the number of susceptible individuals at sea should be a subject for further studies. Similarly, the mechanism by which fish gain resistance to IPN relative to age should also be addressed.

In terms of structural layout, it is interesting to note that P217T, T221A A247T, N252V, S281T, D282N, and E319A are located on the surface loops of the VP2-HVR (Figure 4). Based on observations made from our previous studies as well as observation made by other scientists, residues strategically located on the exterior surface of the HVR of the VP2 capsid significantly influence the virulence and antigenic properties of IPNV (Santi et al., 2004; Song et al., 2005; Coulibaly et al., 2010; Gadan et al., 2013). Although we did not determine the exact mechanism on how different amino acids found on the VP2-HVR influence the clinical and subclinical conditions of IPNV, studies done for other viruses have shown that motifs with high binding potential lead to reduced efficiency on the release of viruses from bound cell receptors resulting in persistent or subclinical infections (Palese et al., 1974; Bauer et al., 1995; Liu et al., 1995). On the contrary, motifs with low binding avidity result in quick virus release resulting in rapid replication culminating in clinical disease (Palese et al., 1974; Bauer et al., 1995; Liu et al., 1995). In our previous studies (Gadan et al., 2013), we observed that having a Threonine at position 221 on the VP2 capsid of IPNV led to reduced virus replication and yet a mutation to an alanine on the same position led to >1000-fold increase in virus replication. It is highly likely that a mutation from threonine, which has a high binding affinity for cell receptors (Betts and Russell, 2003), to an alanine which is less reactive reduces the binding avidity of the virus to cell receptors leading to increased virus release and replication. Therefore, it is likely that differences in the binding avidity of different residues found on the VP2-HVR could account for differences in clinical and subclinical forms of IPNV infections.

The absence of virus at brood stations and its isolation in different fish groups in fresh- or seawater sites (Figure 2) demonstrates that as already mentioned, fresh and seawater sites targeted in this study are contaminated with IPNV. This is consistent with a previous report and supports the view that both fresh and seawater sites are important sources of virus infections for fish (Murray et al., 2003). Furthermore, the increase in prevalence of the virus in fish groups from fresh to seawater (Figure 2) is in agreement with others that virus contamination in seawater is on the increase (Murray et al., 2003). The results also suggest that brood stations are not important sources of IPNV and the testing program used may be a useful means of maintaining the clean status in the supply of breeding materials. In contrast, the absence of IPNV and consequently IPN outbreaks in at least one fish group throughout this study suggests that not all fresh- and seawater sites are contaminated or it is possible to reduce/eradicate IPNV at rearing sites.

The two clades in the phylogenetic tree of the isolates in this study fit very well with the clinical symptoms that the virus induces in Atlantic salmon. Clade 1 was associated with isolates causing overt disease while Clade 2, subclinical infections (Figure 3). Furthermore, the clustering together of isolates from hatcheries and corresponding seawater sites of the same fish groups offers support to the understanding that seawater infections are a recurrence of freshwater infections (Roberts and Pearson, 2005). The clustering together of isolates from one site, renders support to the theory of the existence of “house strains.” Finally, it is noteworthy that the findings in this report relate to Norwegian isolates of IPNV. In Ireland and Scotland, IPNV isolates with P_217_T_221_ motifs were associated with clinical outbreaks (Bain et al., 2008; Ruane et al., 2009, 2015). The explanation for this contradiction is not easy to provide although an interesting subject for future research. Most likely other factors besides virulence as it relates to the virulence motif of IPNV play a role in the outcome of infections.

## Author contributions

Conceived and designed the experiments: SM, ØE, TJ, GR. Performed the experiments: TJ, SM, HM. Contributed reagents/materials/analysis tools: ØE, GR. Analyzed results, discussed and wrote the paper: SM, TJ, GR, HM, ØE

### Conflict of interest statement

ØE, GR, TJ, and SM. 2014. IPN vaccine. United States Patent no. 20140072591. Washington, DC: U.S. Patent and Trademark Office. The other author declares that the research was conducted in the absence of any commercial or financial relationships that could be construed as a potential conflict of interest.
